# PoolParty: streamlined design of DNA sequence libraries in Python

**DOI:** 10.64898/2026.04.06.716802

**Published:** 2026-04-09

**Authors:** Zhihan Liu, Aidan Cordero, Justin B. Kinney

**Affiliations:** 1 Simons Center for Quantitative Biology, Cold Spring Harbor Laboratory, 1 Bungtown Rd., Cold Spring Harbor, New York 11724, United States

**Keywords:** sequence design, library design, massively parallel reporter assay, deep mutational scanning, multiplex assay of variant effect, genomic AI, in silico experiments, Python software, directed acyclic graph

## Abstract

Computationally designed DNA sequence libraries are essential components of many high-throughput assays. They are also increasingly used in silico to analyze genomic AI models. Designing these libraries, however, remains tedious and error-prone. Here we describe PoolParty, a Python package that streamlines the design of complex oligo pools using a simple but flexible API. In PoolParty, each library is represented by a computational graph that can be specified in just a few lines of code. Over 50 built-in operations cover nucleotide- and codon-level mutagenesis, motif insertion, barcode generation, and more. PoolParty also provides “design cards” detailing how each sequence was generated.

## Background

Designed libraries of DNA sequences (oligonucleotide pools) have become essential tools in functional genomics. In massively parallel reporter assays (MPRAs), complex libraries of DNA sequences are routinely used to measure the regulatory activities of tens of thousands of candidate regulatory elements or their variants in a single experiment [1–13]. In deep mutational scanning (DMS) experiments, coding sequences that have been either systematically or randomly mutagenized are commonly used to map protein fitness landscapes [14–20]. Oligo pools can also be used in high-throughput biochemical assays, such as measurements of transcription factor specificity [21–27]. These are all examples of multiplex assays of variant effect (MAVEs), a large and expanding family of high-throughput methods that quantitatively link sequence to function at scale [28–30]. Designed sequence libraries are also used beyond the wet lab: they are increasingly employed in silico to probe the behavior of genomic AI models [31–35] and to augment genomic AI training data [36].

Computationally designing these libraries is straightforward in principle, but in practice it is often tedious and prone to error. Typical applications require oligo pools comprising multiple types of variant sequences. A DMS library, for example, might combine exhaustive single-amino-acid substitutions, sampled higher-order mutants, multiple copies of wild-type and control sequences, and barcodes—each generated by a different procedure and subject to different coverage requirements. An MPRA library might tile multiple transcription factor binding sites across a regulatory element in all possible permutations and orientations, then assign each variant multiple barcodes. Coordinating this kind of combinatorial logic in a one-off script quickly becomes unwieldy, and often produces errors that are hard to catch.

Existing tools can help with parts of this process, but none provide a unified framework for library design. General-purpose sequence toolkits [37–39] can manipulate individual sequences but have no notion of a variant library as a structured object. Consequently, users must write custom scripts to handle the combinatorial logic themselves. Several assay-specific tools automate library design for particular experimental formats. For example, VaLiAnT [40] generates exhaustive single-position variant libraries for DMS and saturation genome editing, including single-nucleotide substitutions, alanine scans, and in-frame deletions. For MPRAs, MPRAnator [41] provides modules for motif placement, SNP design, and negative control generation [see also 42]. However, each of these tools is tied to a specific assay type and does not support combinatorial designs or mixed mutagenesis strategies. While some annotate each variant with the specific mutations it carries, none record the broader design parameters governing how each sequence was generated—parameters that can serve directly as covariates in downstream analyses.

Here we present PoolParty, a Python package that enables the streamlined design of complex DNA sequence libraries. In PoolParty, libraries are represented as objects called *Pools*, while individual steps in the sequence generation process are called *Operations*. Using a concise and transparent syntax, users chain together Operations into a directed acyclic graph (DAG)—an approach inspired by deep learning frameworks—that yields the desired Pool. The DAG describes the high-level logic used to generate sequences, while the procedural details and bookkeeping are handled internally. Importantly, no sequences are generated until the user requests them, so users can explore and test multiple design options without triggering expensive computations. When sequences are generated, each is automatically assigned an informative name and paired with a *design card* that records the specific procedures used to construct it. We demonstrate PoolParty through three example applications: a DMS library for protein GB1, an MPRA library probing transcriptional regulatory grammar, and an in silico experiment characterizing SpliceAI [43], in which design cards provide the covariates for surrogate modeling.

## Results

### Pools and Operations

PoolParty is built around two core abstractions: Pools and Operations. Pools represent collections of sequences—either the final library that the user wishes to generate, or intermediate sets of sequences out of which the final library is constructed. Operations represent the steps used to create Pools. When designing a library, users chain together multiple Operations into a DAG that yields the desired Pool, called the *root Pool*. Sequences are then generated on demand from the root Pool. By delaying sequence generation until it is explicitly requested, users can test many different library designs before committing to generating the full library. For example, users can inspect a few sampled sequences from each one of multiple proposed designs.

**Fig. 1.**
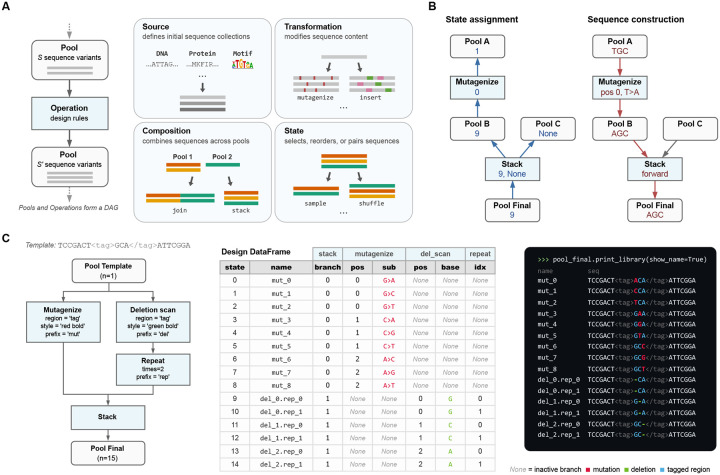
PoolParty builds libraries from Pools and Operations. **(A)** Pools represent collections of sequences, and Operations generate new Pools based on their inputs (left). Users chain Operations into a directed acyclic graph (DAG) that specifies the desired library. Operations fall into four functional categories: source, transformation, composition, and state (right). **(B)** Sequence generation proceeds in two steps: state assignment and sequence construction. For each desired sequence, PoolParty passes to the root Pool a number (called the *state*) that specifies which sequence to generate. During state assignment (left), this state is propagated backward through the DAG, resolving the internal state of every Operation along the way. During sequence construction (right), each Operation generates and combines sequence fragments according to its assigned state, producing the final sequence at the root Pool. When multiple Pools are combined by stack, only one parent is active for a given state; inactive branches receive no state (None) and are shown as grey arrows. **(C)** Example workflow. The template sequence contains a user-specified region (called “tag”) that is targeted for both mutagenesis and deletion scanning. Left: the DAG specifying the sequence library. Center: the design card DataFrame returned by the DAG. Right: styled sequence output, with colors indicating mutations (red), deletions (green), and the tagged region (blue).

PoolParty provides over 50 built-in Operations that users can choose from when constructing DAGs. Each Operation takes zero or more Pools as input and returns exactly one Pool as output. It is useful to think of Operations as falling into four functional categories (Fig. 1A): source, transformation, composition, and state Operations. *Source Operations* create the initial Pools from which all downstream Pools are constructed. The Pools they create may represent user-specified sequences, DNA sequences generated using position weight matrices (PWMs) or IUPAC motifs, random k-mers, barcodes, and so on. *Transformation Operations* modify sequence content, e.g., generating sequence variants through point mutations, insertions, deletions, recombination, or shuffling. Specialized codon-aware Operations are provided for transformations that operate on open reading frames. *Composition Operations* combine sequences from multiple parent Pools, either by concatenating parent sequences end-to-end (the join Operation) or by merging multiple Pools into one Pool (the stack Operation). *State Operations* affect which sequences are in a Pool and how they are ordered, and include methods to select, reorder, filter, and replicate sequences.

### Sequence generation

To generate a sequence library, PoolParty iterates through a series of *states*. Each state is an integer that, together with a random seed, uniquely determines a sequence. The generation of each individual sequence proceeds in two steps, state assignment and sequence construction (Fig. 1B), a process that begins when the corresponding state is passed to the root Pool. State assignment involves a *backward pass* through the DAG. Each Pool passes the state it receives to its upstream Operation. Each Operation then decomposes this state into an *internal state* (which it keeps) and zero or more *component states*, each of which is passed to a distinct upstream Pool. Sequence construction then occurs during a *forward pass* through the DAG: each Operation constructs a sequence based on its internal state and the sequences received from the upstream Pools, then passes this sequence to the downstream Pool. The final constructed sequence is then received from the root Pool and recorded.

### Operation modes

The specific way that each Operation interprets its internal state is determined by its *mode*.

- In *sequential mode*, the Operation exhaustively enumerates a finite set of sequence designs. For example, when mutagenize is instructed to generate single-nucleotide mutations in sequential mode, it yields one variant for each possible character change at each target position. Its internal state specifies which mutation to apply at which position.
- In *random mode*, the Operation samples sequence designs stochastically. For example, when mutagenize operates in random mode, it randomly selects which positions to mutate and which mutations to introduce. Its internal state, combined with a master seed, determines the specific random draws, ensuring reproducibility.
- In *fixed mode*, there is no internal state. Rather, output sequences are uniquely determined by the input sequences. One example is join, which concatenates its input sequences without modification.

### StateTracker

The way each Operation decomposes the state it receives during the backward pass is designed to reverse the way it constructs sequences during the forward pass. For most Operations, the set of possible output states is the Cartesian product of the possible internal states and the possible states of each input Pool. In such cases, the Operation uses mixed-radix decomposition to split the recieved state into its component states. The stack Operation is different: its output states are the disjoint union of its input Pool states. The stack Operation therefore uses the state it receives to select which input Pool to use, and to instruct this Pool (but not the other Pools) which sequence to return. Keeping track of these rules across a complex DAG requires specialized bookkeeping. We therefore developed a back-end package called StateTracker that automates state composition and decomposition during forward and backward passes through the DAG.

### Sequence regions

One may want to perform different Operations on different parts of a sequence. To facilitate this, PoolParty lets users mark regions of interest within sequences using XML-style tags. PoolParty supports both opening/closing tag pairs (e.g., AAA<cre>CCCGGG</cre>TTT) and self-closing tags (e.g., ACGT<ins/>ACGT). Operations can then be instructed to act only on a specified region, as illustrated in Fig. 1C. Tags persist through the DAG and remain valid even when upstream Operations change the content of a region. Multiple Operations can thus target modifications to the same region in series.

### Sequence metadata

It is often useful to record how each sequence within a library was constructed. PoolParty provides three complementary ways to do this (Fig. 1C).

- *Design cards* record the specific changes applied to each sequence by each Operation. Users specify which Operations and Operation-specific variables to track. As each sequence is generated, the root Pool returns it together with a design card that records the tracked variable values for that sequence. PoolParty compiles these into a DataFrame in which each row contains a generated sequence and its design card.
- *Sequence names* summarize at a glance how each sequence was constructed. Users assign a prefix to each Operation they wish to track. PoolParty then pairs each prefix with the corresponding Operation’s internal state to form a token (e.g., mut_03), and concatenates these tokens to form the sequence’s name. For example, wt.mut_03.bc_01 might denote a wild-type sequence with mutation variant number 3 and barcode number 1.
- *Sequence styling* allows sequences to be annotated with colors and formatted text that reflects each one’s construction history. For example, mutagenize can be instructed to mark the positions it mutates in bold yellow text, making these positions stand out from the rest of the sequence. This formatting is accomplished using ANSI escape codes, so that output renders correctly in command-line terminals, Jupyter notebooks, and other environments that support basic text formatting. These styles combine in a natural manner as sequences propagate through the DAG. For example, a mutated position can be underlined even if it occurs within a sequence region that is already colored blue.

### Example 1: DMS library for protein GB1

As a first example, we use PoolParty to reconstruct and extend the library used in a DMS experiment on protein GB1 by Olson et al. [17], adding random higher-order mutants and wild-type replicates to the original design. Their library contained the wild-type GB1 coding sequence, all single amino acid substitutions (excluding the start codon), and nearly all possible pairs of amino acid substitutions. This library is substantially larger than most DMS libraries, containing over half a million variants. Its inclusion of so many pairwise variants has helped it become a benchmark dataset in the quantitative modeling of protein sequence-function relationships [44–47].

**Fig. 2.**
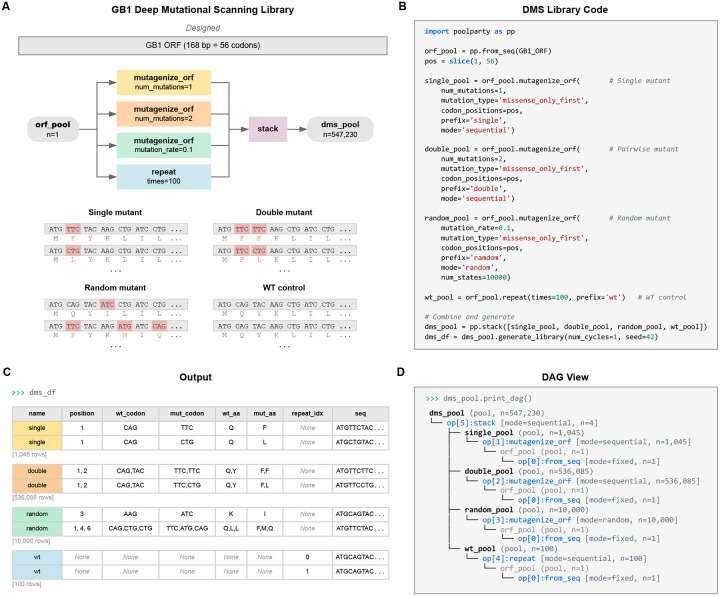
A deep mutational scanning (DMS) library for protein GB1. **(A)** DAG and representative sequences for each of the four library components: replicates of the wild-type sequence, all single amino acid substitutions, all pairwise amino acid substitutions, and random higher-order mutants. Mutated codons are highlighted with amino acid translations shown beneath. **(B)** Python code implementing the DAG. **(C)** Design cards reporting the library component, mutation positions, and amino acid changes for representative variants. **(D)** DAG structure displayed by print_dag().

Fig. 2A illustrates the DAG used to construct this library; the corresponding Python code is shown in Fig. 2B. First, we use the from_seq Operation to define a Pool named orf_pool that represents the wild-type GB1 coding sequence. This Pool is then passed to four separate Operations that define the four library components. The first is a repeat Operation that produces 100 copies of the wild-type sequence. The second and third are mutagenize_orf Operations running in sequential mode: one generates all 1,045 single amino acid substitutions, and the other generates all 536,085 pairwise amino acid substitutions. The fourth is a mutagenize_orf Operation running in random mode, which mutates each amino acid position with 10% probability to generate 10,000 higher-order variants. We note that mutagenize_orf supports multiple codon mutation types; this example uses missense_only_first, which selects the most abundant codon for each amino acid substitution. These four Pools are then merged by stack into the root Pool, yielding a library of 547,230 sequences in total. The design card for each variant (Fig. 2C) reports which component it belongs to, the specific mutations it contains, and the replicate index (for wild-type copies). Users can also inspect the DAG structure by calling the print_dag method of the root Pool (Fig. 2D).

### Example 2: MPRA library for regulatory grammar

MPRA libraries used to study regulatory grammar often vary the positions and orientations of transcription factor binding sites (TFBSs) [3, 5, 48–52]. For example, Georgakopoulos-Soares et al. [52] studied the impact of order and orientation on gene expression by testing over 200,000 sequences containing all pairwise and triplet arrangements of 18 TFBSs. Designing such libraries requires substantially more complex combinatorics than standard DMS libraries do. PoolParty, however, handles this complexity without making additional demands on the user.

**Fig. 3.**
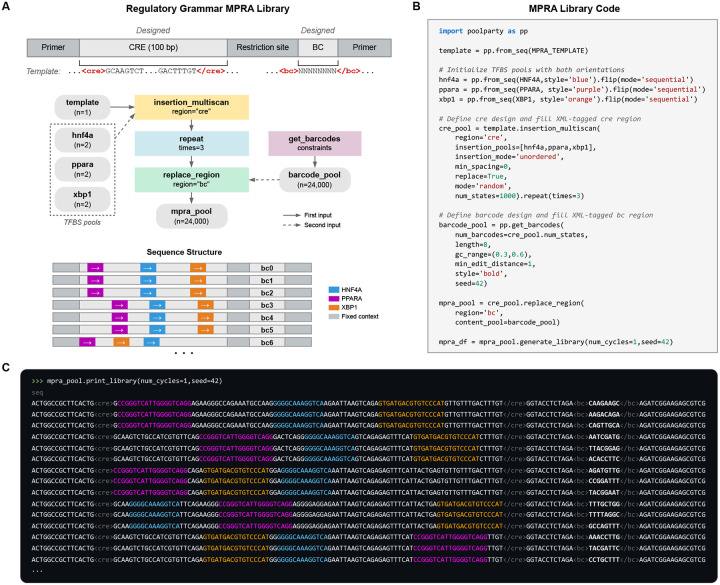
A massively parallel reporter assay (MPRA) library for probing regulatory grammar. **(A)** Library schematic showing TFBS placements across a 100-bp CRE in representative variants, each linked to three different barcodes. **(B)** Python code for library construction. TFBS Pools are initialized with forward sequences and augmented with the flip Operation to include both orientations. **(C)** Output of print_library. TFBSs are colored by identity (HNF4A blue, PPARA purple, XBP1 orange); barcodes are shown in bold.

As a second example, we designed an MPRA library representing various arrangements of binding sites for three liver-enriched TFs—HNF4A, PPARA, and XBP1 (Fig. 3A,B). Following the oligo layout of Melnikov et al. [3], we start by creating a Pool representing a template sequence that contains a putatively inert 100 bp CRE region (<cre>) and a barcode region (<bc>). Next, we create a Pool representing the binding site for HNF4A. This Pool is then passed to the flip Operation, which yields both orientations of the site (forward and reverse complement). Pools for PPARA and XBP1 are created similarly. The template Pool and three TFBS Pools are then passed to the insert_multiscan Operation, which creates 10,000 sequences by inserting the TFBSs at random positions, chosen to avoid overlaps, within the <cre> region of the template. Each sequence is then replicated three times using the repeat Operation. Finally, the get_barcodes Operation is used to generate barcodes that are inserted into the <bc> region of the sequence. The result is a library of 30,000 sequences (Fig. 3A).

Colored text helps users inspect the architecture of the resulting sequences. After the template Pool is created, the stylize Operation is used to shade the <cre> region gray. Each TFBS Pool is also assigned a distinct color when it is created (HNF4A blue, PPARA purple, XBP1 orange); this is specified by the style parameter passed to from_seqs. Barcodes are similarly rendered in bold text (Fig. 3B). These styles compose as sequences pass through the DAG, so that when print_library is called (Fig. 3C), the position, order, and orientation of each binding site are immediately visible.

### Example 3: Surrogate modeling of SpliceAI predictions

Designed sequence libraries are also used to study genomic AI models [31–33, 35]. First, an in silico experiment is carried out in which the AI model scores all sequences in a library. The results are then analyzed to characterize model behavior. One useful way of analyzing these synthetic data is *surrogate modeling*, in which a mathematically simple model—one whose structure and parameters are directly interpretable—is fit to the predictions.

In addition to streamlining the design of sequence libraries, PoolParty facilitates in silico experiments on genomic AI models in two ways. First, because sequence design is separated from sequence generation, new sequences can be generated on demand as the experiment proceeds. Second, the design card that PoolParty returns with each sequence provides metadata that can be used directly as covariates in downstream analyses, including surrogate modeling.

**Fig. 4.**
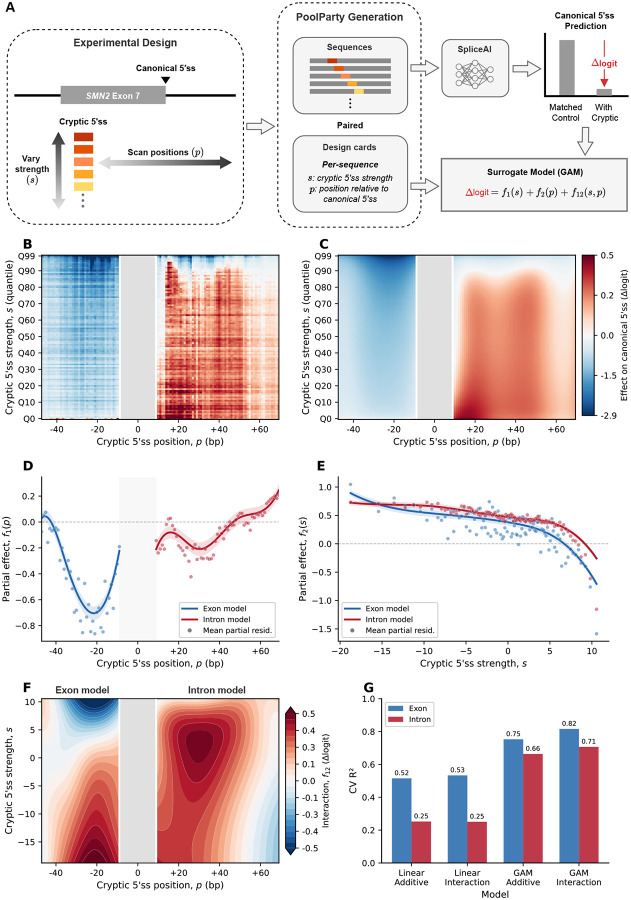
Surrogate modeling of SpliceAI predictions. **(A)** Experimental design. Cryptic 5’ss 9-mers of varying MaxEntScan strength (*s*) are inserted at varying positions (*p*) flanking the canonical 5’ss of SMN2 exon 7. Each variant is paired with a control in which the splice-site GT dinucleotide is disrupted (GT→GA). PoolParty records *s* and *p* in design cards. The effect of each insertion on SpliceAI predictions at the canonical 5’ss is quantified as Δlogit and modeled as a function of *s* and *p* using a generalized additive model (GAM). **(B)** Mean Δlogit across the strength × position grid (100 × 100; each cell averages 20 distinct 9-mers). The grey band marks the canonical 5’ss region. **(C)** GAM predictions (including interaction term), using the same color scale as in B. **(D)** Partial dependence of the position term *f*_1_(*p*), shown separately for exon (blue) and intron (red) models. Shaded regions indicate 95% confidence intervals; points denote mean partial residuals. **(E)** Partial dependence of the strength term *f*_2_(*s*), with conventions as in D. **(F)** Interaction surface *f*_12_(*s,p*) for exon and intron models. **(G)** Block cross-validation *R*^2^ for linear and GAM models, with and without interaction terms.

As a third example, we carried out a surrogate modeling study of SpliceAI [43], a deep learning model of mRNA splicing. We asked how the insertion of a cryptic 5’ splice site (5’ss) near an existing (canonical) 5’ss affects the model’s prediction for canonical site usage. Using the 5’ss of SMN2 exon 7 as the canonical site, we used PoolParty to construct a library in which cryptic 5’ss 9-mers of varying strength were inserted at varying positions on either side of the canonical 5’ss (Methods). The specific values for cryptic site strength and position were recorded in the design cards (Fig. 4A).

Each variant was paired with a matched control in which the cryptic site was disrupted, and the effect of each insertion was quantified as the difference in logit-transformed SpliceAI-predicted probabilities at the canonical site (Δlogit). Generalized additive models (GAMs) were used to separately model the effects of cryptic site insertion at exonic and intronic positions. Each of these two models has three terms, corresponding to the effects of position alone, strength alone, and interactions between these covariates (Fig. 4A).

The GAMs closely reproduced the observed data (held-out *R*^2^ of 0.82 for exonic and 0.71 for intronic insertions; Fig. 4B,C). Cryptic sites on the exonic side suppressed canonical site usage more strongly than those on the intronic side (Fig. 4D), with the effect peaking approximately 20 bp upstream of the canonical 5’ss. This is consistent with the observation that cryptic 5’ss are preferentially depleted near annotated ones on the exonic side [53]. Stronger cryptic sites drove greater suppression in both models (Fig. 4E). The effects of position and strength exhibited substantial interactions (Fig. 4F). Through a comparison to analogous linear models, we observed that the non-linear terms and interaction terms in the GAMs substantially improved predictive performance (Fig. 4G).

## Discussion

We have presented PoolParty, a Python package that replaces the ad hoc scripting commonly used to design oligonucleotide libraries with a declarative, composable, and flexible framework. We demonstrated PoolParty through three use cases: a DMS library for protein GB1; an MPRA library probing transcriptional regulatory grammar; and a surrogate modeling analysis of the effects of cryptic 5’ss on SpliceAI predictions. These examples illustrate the flexibility of PoolParty and its applicability to both experimental and computational studies.

PoolParty was inspired by deep learning frameworks like PyTorch and TensorFlow, in which a high-level API is used to construct a DAG representing the desired neural network. The DAG then enables computation of model gradients via backpropagation. Similarly, in PoolParty, a backward pass through the DAG is used to decompose the state for a requested sequence into the specific Operation internal states needed to construct that sequence during a subsequent forward pass. This state-decomposition mechanism is a core innovation of PoolParty; it is what allows users to design complex oligo pools and test them before generating the full sequence library.

We also highlight PoolParty’s design cards. Design cards allow the parameters governing each sequence’s construction to serve directly as covariates in downstream analyses, eliminating the need for post hoc sequence parsing. In the SpliceAI example, the cryptic splice-site strength and position recorded in each design card provided the covariates used for surrogate modeling. We expect design cards to be increasingly useful as in silico experiments on genomic AI models become more common.

In contrast to assay-specific tools such as VaLiAnT [40] and MPRAnator [41], PoolParty provides a single framework that supports DMS libraries, MPRA libraries, and library designs that combine elements of both. Its DAG-based architecture lets users freely compose arbitrary sequence generation and transformation operations. Because the DAG encodes the full construction logic, structured design-card metadata are generated automatically as a byproduct.

PoolParty was designed to address a focused computational problem: the streamlined design of DNA sequence libraries. As such, it has several limitations. PoolParty is not a sequence optimization tool, e.g., for optimizing codon usage [54], satisfying synthesis constraints on individual sequences [55], or designing sequences guided by machine learning models [56]. Nor does it address the problem of physically constructing DNA sequence libraries, e.g., by designing mutagenic primers [57]. We expect that PoolParty can in many cases be productively paired with such complementary tools.

## Conclusions

PoolParty should substantially ease the design of DMS experiments, MPRAs, and other multiplex assays of variant effect. As genomic AI models continue to advance, PoolParty will also help researchers systematically probe and interpret the behavior of these models. Moreover, because PoolParty’s composable design makes it straightforward to define new Operations, the framework can readily be extended to support novel assay types and analysis strategies as they emerge.

## Methods

### Implementation and extensibility

PoolParty requires Python 3.10 or later, is designed to have minimal dependencies, and is distributed via PyPI. Documentation, tutorials, and example notebooks are available on the ReadTheDocs and GitHub project websites. PoolParty is also extensible: users can create new Operation types by subclassing the Operation base class and implementing a few required methods. Custom Operations automatically inherit support for state tracking, design card generation, and visualization, allowing researchers to add domain-specific functionality without reimplementing core infrastructure.

### SpliceAI surrogate analysis

This section provides technical details for the surrogate modeling analysis presented in Results. Using the human 5’ss position weight matrix [58], we sampled 9-mers representing cryptic 5’ss sequences and scored them with MaxEntScan [59]. Sequences were binned into 100 MaxEntScan score (hereafter, strength) quantiles, and 20 sequences were drawn from each bin (2,000 total). Each 9-mer was inserted at 100 positions flanking the canonical 5’ss of SMN2 exon 7 and paired with a matched control in which the GT dinucleotide was disrupted (T>A at +2; Fig. 4A).

We predicted SpliceAI 5’ss probabilities at the canonical site using the five-model ensemble of Jaganathan et al. [43] with ±5 kb of genomic context (GRCh38). For each library-control pair, we computed the difference in logit-transformed predictions at the canonical 5’ss. These per-sequence values were averaged within each cell of the resulting 100 × 100 position-by-strength grid (Fig. 4B).

We fit generalized additive models (GAMs) to the cell means using pyGAM [60], with smooth terms for position, strength, and their interaction. Models were weighted by the inverse variance of each cell mean. Exonic and intronic regions were modeled separately. Model performance was evaluated by checkerboard cross-validation of the grid.

### LLM disclosure

Assistance from LLMs, Claude Opus 4.5 and 4.6 in particular, was used across all facets of this project.

## Data Availability

PoolParty is available as an open-source Python package under the MIT license. Source code is available at http://github.com/jbkinney/poolparty-statetracker. Documentation is provided at http://poolparty.readthedocs.io. The package can be installed via pip and is compatible with Python ≥ 3.10. A static release of the version described in this manuscript is deposited at DOI 10.5281/zenodo.19445098.
